# Coenzyme Q10-Loaded Albumin Nanoparticles Protect against Redox Imbalance and Inflammatory, Apoptotic, and Histopathological Alterations in Mercuric Chloride-Induced Hepatorenal Toxicity in Rats

**DOI:** 10.3390/biomedicines11113054

**Published:** 2023-11-14

**Authors:** Shimaa S. Ramadan, Farah A. El Zaiat, Engy A. Habashy, Mostafa M. Montaser, Habeba E. Hassan, Shahinaz S. Tharwat, Manal El-khadragy, Ahmed E. Abdel Moneim, Gehad E. Elshopakey, Ahmed M. A. Akabawy

**Affiliations:** 1 Biochemistry Sector, Chemistry Department, Faculty of Science, Helwan University, Cairo 11795, Egypt; 2 Molecular Biotechnology Sector, Chemistry Department, Faculty of Science, Helwan University, Cairo 11795, Egypt; 3 Department of Biology, College of Science, Princess Nourah bint Abdulrahman University, P.O. Box 84428, Riyadh 11671, Saudi Arabia; mfelkhadragy@pnu.edu.sa; 4 Zoology and Entomology Department, Faculty of Science, Helwan University, Cairo 11795, Egypt; 5 Department of Clinical Pathology, Faculty of Veterinary Medicine, Mansoura University, Mansoura 35516, Egypt; 6 Department of Biochemistry and Molecular Biology, Faculty of Pharmacy, Helwan University, Cairo 11795, Egypt

**Keywords:** mercuric chloride, coenzyme Q10, nanoparticles, hepatorenal toxicity, oxidative stress, inflammation, apoptosis

## Abstract

Exposure to mercuric chloride (HgCl_2_), either accidental or occupational, induces substantial liver and kidney damage. Coenzyme Q10 (CoQ10) is a natural antioxidant that also has anti-inflammatory and anti-apoptotic activities. Herein, our study aimed to investigate the possible protective effects of CoQ10 alone or loaded with albumin nanoparticles (CoQ10NPs) against HgCl_2_-induced hepatorenal toxicity in rats. Experimental animals received CoQ10 (10 mg/kg/oral) or CoQ10NPs (10 mg/kg/oral) and were injected intraperitoneally with HgCl_2_ (5 mg/kg; three times/week) for two weeks. The results indicated that CoQ10NP pretreatment caused a significant decrease in serum liver and kidney function markers. Moreover, lowered MDA and NO levels were associated with an increase in antioxidant enzyme activities (SOD, GPx, GR, and CAT), along with higher GSH contents, in both the liver and kidneys of intoxicated rats treated with CoQ10NPs. Moreover, HgCl_2_-intoxicated rats that received CoQ10NPs revealed a significant reduction in the hepatorenal levels of TNF-α, IL-1β, NF-κB, and TGF-β, as well as an increase in the hepatic level of the fibrotic marker (α-SMA). Notably, CoQ10NPs counteracted hepatorenal apoptosis by diminishing the levels of Bax and caspase-3 and boosting the level of Bcl-2. The hepatic and renal histopathological findings supported the abovementioned changes. In conclusion, these data suggest that CoQ10, alone or loaded with albumin nanoparticles, has great power in reversing the hepatic and renal tissue impairment induced by HgCl_2_ via the modulation of hepatorenal oxidative damage, inflammation, and apoptosis. Therefore, this study provides a valuable therapeutic agent (CoQ10NPs) for preventing and treating several HgCl_2_-induced hepatorenal disorders.

## 1. Introduction

The industrial contamination of the environment with metal compounds is becoming a serious issue [1]. The most pervasive environmental contaminant among all heavy metals is mercury, which is also employed in industrial, pharmaceutical, agricultural, and other industries [2]. Mercury is ranked as the third most harmful heavy metal in the world by the Agency for Toxic Substances and Disease Registry (ATSDR) [3]. Exposure to methyl mercury (MeHg) and inorganic mercury (mercuric chloride, HgCl_2_), either accidental or occupational, has the potential to induce substantial organ damage in both humans and animals, especially in areas with high levels of food, water, and air pollution [4].

Mercuric chloride is known to be the most hazardous form of metal because it readily forms organomercury compounds with proteins [5,6]. It is widely documented that inorganic mercury has genotoxic [7], hepatotoxic [2], neurotoxic [6], nephrotoxic [8], hematologic [9], and reproductive toxic [10] effects. Various mechanisms underlie the pathophysiology of hepatorenal mercury toxicity, among which is excessive free radical generation and the disruption of antioxidant system homeostasis, which results in oxidative damage to hepatic and renal cells [11]. It was also declared that chronic liver injury is mediated by HgCl_2_ by triggering the transcription of NF-κB/p53 signaling, which, in turn, causes significant inflammatory and apoptotic responses [12]. Furthermore, previous reports have described hepatic and renal cell death as a result of HgCl_2_ exposure in rats [13,14]. Therefore, it is urgent to find a remedy to alleviate the harmful effects of HgCl_2_ with minimal side effects.

Coenzyme Q10 (CoQ10) is a natural antioxidant that is produced by living organisms in the mitochondrial matrix intermembrane [15]. It can also be found in fruits like strawberries, oranges, and apples, as well as vegetables like broccoli, spinach, soybeans, palm oils, canola, almonds, and legumes [16,17]. CoQ10 is 1, 4-benzoquinone, with the letter “Q” standing for the quinone group and the number “10” referring to the isoprenyl units at the end. CoQ10 is mostly found in organs with high energy needs, such as the liver, kidney, and heart, where there are many mitochondria [18]. It can act as a redox agent that significantly contributes to the movement of electrons through the mitochondrial electron transport chain, which leads to the production of ATP [18]. Additionally, CoQ10 has strong free-radical-scavenging abilities that support the maintenance of the mitochondrial membrane potential, as well as the prevention of DNA damage, protein oxidation, and LPO; thus, it can protect cellular functions from oxidative stress [19,20]. Also, CoQ10 has anti-inflammatory and anti-apoptotic activities [18], in addition to boosting the production of critical antioxidants [21].

Recent research has shown that encapsulation-based drug delivery systems can boost the therapeutic effectiveness of pharmaceuticals by increasing their stability in vivo, absorption by cells and tissues, and accumulation at the target [22,23]. Examples of effective nano-formulations for stabilizing synthetic pharmaceutical plant extracts and their active components are protein nanoparticles (NPs). Among the variety of proteins widely used to prepare nanospheres and nanocapsules is bovine albumin serum (BSA) [24]. BSA is the most prevalent protein in blood plasma and has been cited as one of the most promising drug carriers, as it is biodegradable, non-immunogenic, water-soluble, non-toxic, and not detrimental to living tissues [25]. BSA exhibits emulsifying, foaming, and gelation capabilities in addition to its capacity to self-associate [26]. It is a suitable option for encapsulating bioactive substances that are both hydrophilic and hydrophobic, such as vitamins, polyunsaturated fatty acids, carotenoids, and phenolic compounds [27].

The analysis of the previous literature revealed that there are no reports that have evaluated the potency of CoQ10-loaded albumin nanoparticles (CoQ10NPs) against HgCl_2_ toxicity in a rat model. Accordingly, the current study was conducted to elucidate the potential protective properties of CoQ10NPs against HgCl_2_-mediated hepatorenal toxicity. The prospective mechanisms implicated in these effects are also demonstrated, including oxidative status, inflammation, apoptosis, and histological alterations. These findings offer new perspectives for developing safe and effective strategies for mercury detoxification.

## 2. Materials and Methods

### 2.1. Chemicals

The powder forms of mercury chloride (HgCl_2_, 99% purity) and bovine albumin serum (BSA) were obtained from Sigma Aldrich (Hamburg, Germany). Coenzyme Q 10 was purchased from MEPACO (Cairo, Egypt) in the form of firm gelatin capsules (30 mg).

### 2.2. Preparation and Characterization of CoQ10NPs

The preparation of the CoQ10-loaded albumin nanoparticles (CoQ10NPs) was based on the emulsification–solvent-evaporation process previously conducted by Kianfar [28] and Elzoghby et al. [29]. The final concentration of CoQ10 was achieved by gradually dissolving it in ethyl acetate (20 mg/mL). CoQ10 solutions were combined with bovine serum albumin dispersion, and a high-speed dispersion machine was used to shear the mixture for two minutes at 12,000 r.m.p. Next, a high-pressure homogenizer was used to homogenize the mixture twice at 60 MPa. After forming nanoparticle suspensions, ethyl acetate was removed by rotary evaporation in a 35 °C water bath (Nanbei Instrument Limited, RE6000, Zhengzhou, China). Distilled water was then added in an amount equal to the volume of lost ethyl acetate. Throughout the entire process, the samples were shielded from light using aluminum foil to prevent CoQ10 degradation. The final mass ratio of albumin to CoQ10 was 20:1, and the final concentration of CoQ10 in the system was 10 mg/mL. The generated CoQ10NPs were analyzed using FTIR (Fourier transform infrared) analysis and the zeta-potential distribution to ensure consistent materials. In addition, a Zetasizer was used to measure the particle size distribution. Prior to delivery, the various CoQ10NP dosages were sonicated in normal saline for 10 min to ensure a uniform distribution so they could be easily absorbed by the systemic circulation.

### 2.3. Animals, Treatment Protocol, and Sampling

Forty adult male Western albino rats, weighing between 120 and 150 g, were obtained from VACERA company (Giza, Egypt). The rats were kept in polypropylene cages with controlled light–dark cycles (12 h:12 h), temperature (22 ± 2 °C), and humidity (50 ± 10%), and they were given access to a normal balanced rodent meal with water ad libitum. The study methodology and all experimental techniques were approved by the Committee of Research Ethics for Laboratory Animal Care, Department of Zoology and Entomology, Faculty of Science, Helwan University (Cairo, Egypt; Permit Number: HU2022/Z/AES0922-01).

Following the two-week acclimatization period, the rats were distributed equally into five groups, each consisting of eight rats. Group I (control) orally received 1% carboxymethylcellulose (100 μL), and after one hour, the rats were intraperitoneally (i.p.) injected with saline (100 μL). Group II (CoQ10) was gavaged with 10 mg/kg BW of coenzyme Q10 [30]. Group III (HgCl_2_) intraperitoneally received 5 mg HgCl_2_/kg body weight (BW) dissolved in saline (three times/week), as previously reported by El-Desoky [31]. Group IV (CoQ10-HgCl_2_) received CoQ10 (10 mg/kg BW/orally) one hour before the injection of HgCl_2_ (5 mg/kg BW/i.p.). Group V (CoQ10NPs-HgCl_2_) received 10 mg/kg BW of coenzyme Q10-loaded albumin nanoparticles 60 min before the intraperitoneal injection of HgCl_2_.

CoQ10 powder was prepared in 1% carboxymethylcellulose before being administered through a stomach tube [32], while CoQ10NPs were uniformly distributed in normal saline (0.9% *w*/*v*) for 15 min via sonication before oral administration [33]. CoQ10 and CoQ10NPs were given as a single dose per day over the course of 14 days. Within 24 h following the last treatment, the rats were euthanatized and sacrificed. Blood samples were collected and left at room temperature for 20 min to clot. They were centrifuged at 3000× *g* for 10 min to separate the serum. The serum samples were stored at −20 °C for further analysis. The liver and kidney tissues were immediately removed and separated into two pieces. The first portion of the liver and kidney tissues was homogenized (10% *w*/*v*) in Tris-HCl buffer (50 mM; pH 7.4) and centrifuged at 5000× *g* for 10 min. The supernatants were isolated and stored at –80 °C for biochemical studies. For histological analysis, the second portion of each tissue was placed in a 10% neutral formaldehyde solution.

### 2.4. Serum Liver and Renal Markers

Serum ALT (catalog no. AL 1031), AST (catalog no. AS 1061), urea (catalog no. UR 2110), and creatinine (catalog no. CR 1251) were quantitated spectrophotometrically using diagnostic kits obtained from Laboratory Biodiagnostics Company (Cairo, Egypt). Additionally, an ELISA kit (catalog no. 80684) acquired from Crystal Chem company (Elk Grove Village, IL, USA) was used to assess kidney injury molecule-1 (Kim-1). Briefly, KIM-1 present in the sample binds to antibodies bound to the surface of the microplate. After washing, the labeled antibodies are added and form a complex on the surface. The concentration of KIM-1 was measured based on color intensity.

### 2.5. Hepatorenal Oxidative/Antioxidant Status

In hepatic and renal tissues, the lipid peroxidation marker (malondialdehyde; MDA), nitric oxide (NO), superoxide dismutase (SOD), glutathione (GSH), glutathione peroxidase (GPx), glutathione reductase (GR), and catalase (CAT) were colorimetrically measured based on the methodologies of Ohkawa et al. [34], Green et al. [35], Nishikimi et al. [36], Ellman [37], Paglia and Valentine [38], Smith et al. [39], and Aebi [40], respectively. Later, the protein content in the tissue homogenates was measured using the Bradford [41] method to calculate oxidative/antioxidant biomarkers per milligram of protein.

### 2.6. Hepatorenal Inflammatory and Fibrotic Mediators

Specific ELISA kits for rats obtained from MyBioSource company (San Diego, CA, USA) were used to evaluate the hepatic and renal levels of tumor necrosis factor-alpha (TNF-α, catalog no. MBS2507393), interleukin-1 beta (IL-1β, catalog no. MBS825017), nuclear factor kappa (NF-ĸβ, catalog no. MBS287521), transforming growth factor-β (TGF-β, catalog no. MBS160117), and hepatic alpha-smooth muscle actin (α-SMA, catalog no. MBS266620). 

### 2.7. Hepatorenal Apoptotic Markers

B-cell lymphoma-2 (Bcl-2, catalog no. MBS2515143) and Bcl-2-associated X protein (Bax, catalog no. MBS2512405) were assessed following the producer’s approach. ELISA kits purchased from Enzo Life Sciences Company (Farmingdale, NY, USA) were used to measure the caspase-3 activity (catalog no. ADI-907-013) according to the enclosed pamphlet.

### 2.8. Histopathology of Liver and Kidney

Liver and kidney tissues were encased in paraffin wax and sliced into 4–5 μm thick slices using a microtome following 24 h of preservation in 10% neutral formaldehyde. Conventional procedures were used to stain the sections with hematoxylin and eosin (H&E). The prepared slides were viewed with a Nikon Eclipse E200-LED microscope (Tokyo, Japan) at 400× magnification.

### 2.9. Statistical Evaluation

The analyzed data were analyzed and presented as means with standard deviations (SDs) using the Statistical Package for the Social Sciences (SPSS, version 17). All data were tested for normality by performing Levene’s test. The histogram figures were created using the GraphPad Prism data analysis application (version 6.1; GraphPad Software, Inc., San Diego, CA, USA). A one-way analysis of variance (ANOVA) was used to evaluate the variance across groups. A *p*-value of less than 0.05 was regarded to be an acceptable level of significance.

## 3. Results

### 3.1. Coenzyme Q10-Loaded Albumin Nanoparticle (CoQ10NP) Characterization

**Figure 1:**
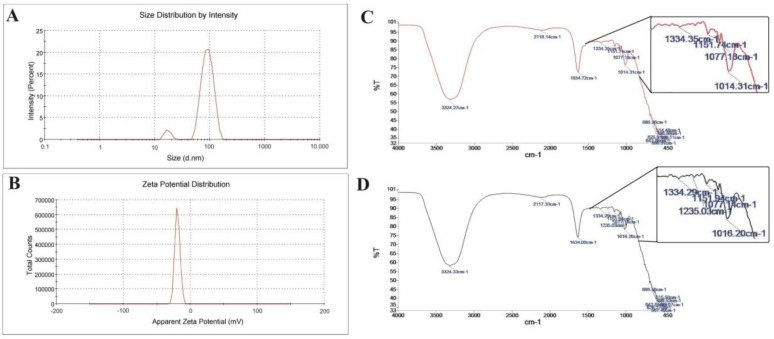
Characterization of coenzyme Q10 coupled to albumin nanoparticles. ((**A**) Upper panel on left): Hydrodynamic diameter of CoQ10NPs measured with Zetasizer. ((**B**) Bottom panel on left): Surface charge of CoQ10NPs expressed as zeta potential. ((**C**) Upper panel on right): FT-IR spectra of CoQ10. ((**D**) Bottom panel on right): FT-IR spectra of coenzyme Q10 coupled to albumin nanoparticles.

As depicted in Figure 1, the particle size of the developed CoQ10NPs is 89.1 ± 35.2 nm. Albumin possesses a molecular weight of 66,500 Daltons (Da), while the molecular weight of CoQ10 is 863.34 Da, and that of CoQ10NPs is 67,363 Da. Moreover, the zeta potential of CoQ10NPs is −19.3 mV; NPs with a ZP less than −30 mV are considered electro-optically stable, as charged particles will repel each other, reducing the chance of their aggregation. Furthermore, the FT-IR of CoQ10 displayed a new bond at 1235.03 cm^−1^, indicating the successful interaction between CoQ10 and albumin. The bands from 1200 to 1350 cm^−1^ revealed an amide region and were attributed to out-of-phase C−N stretching and out-of-phase in-plane N-H bending in the peptide backbone of the proteins (Supplementary data: Table S1).

### 3.2. Effect of CoQ10NPs on Serum Hepatic and Renal Markers upon HgCl_2_ Exposure

**Figure 2:**
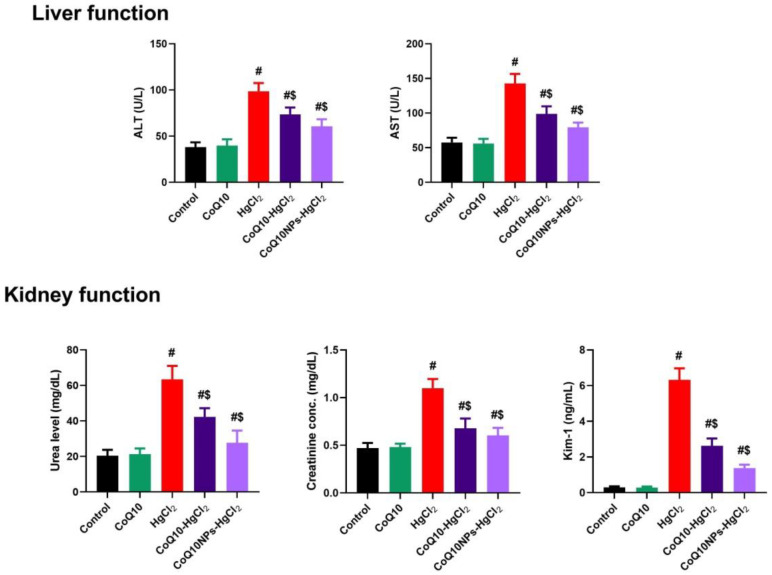
Palliative effects of coenzyme Q10-loaded albumin nanoparticles (CoQ10NPs, 10 mg/kg, orally, for 14 days) on serum hepatic and renal function biomarkers upon exposure to mercuric chloride (HgCl_2_, 5 mg/kg, i.p., three times a week). Data are expressed as mean ± SD (n = 8/group). The symbol ^#^ means a significant difference from the control group, while ^$^ means a significant difference from the HgCl_2_ group at *p*-value < 0.05.

The serum activities of ALT and AST, along with urea, creatinine, and Kim-1, were significantly elevated in the HgCl_2_-intoxicated group parallel to the control group (*p* < 0.05). Meanwhile, the treatment of HgCl_2_ toxicity with CoQ10 alone or conjugated with albumin nanoparticles could significantly lower the aforementioned parameters compared to the HgCl_2_ group (*p* < 0.05), especially in the CoQ10NPs+HgCl_2_ group (Figure 2). These results demonstrate the noteworthy ability of CoQ10NPs to protect against hepatorenal damage mediated by HgCl_2_ toxicity.

### 3.3. Effect of CoQ10NPs on Hepatorenal Oxidative Stress Markers upon HgCl_2_ Exposure

**Figure 3:**
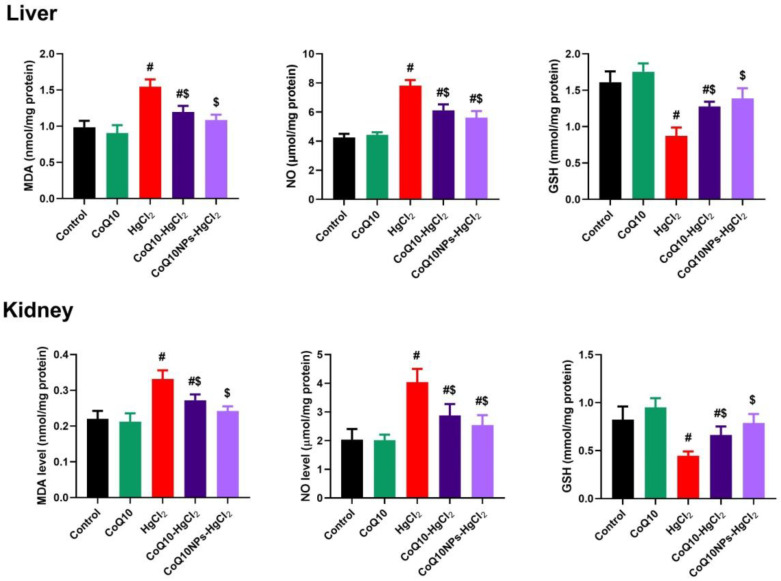
Palliative effects of coenzyme Q10-loaded albumin nanoparticles (CoQ10NPs, 10 mg/kg, orally, for 14 days) on the hepatorenal oxidative stress markers malondialdehyde (MDA), nitric oxide (NO), and superoxide dismutase (SOD) upon exposure to mercuric chloride (HgCl_2_, 5 mg/kg, i.p., three times a week). Data are expressed as mean ± SD (n = 8/group). The symbol ^#^ means a significant difference from the control group, while ^$^ means a significant difference from the HgCl_2_ group at *p*-value < 0.05.

Regarding the oxidative stress markers (Figure 3), higher hepatic and renal levels of lipid peroxide (MDA) and NO accompanied by lower GSH content were detected in HgCl_2_-exposed rats compared to the control (*p* < 0.05). Fortunately, MDA, NO, and GSH were restored in both the liver and kidney by CoQ10 and CoQ10NP compared to the intoxicated untreated group (*p* < 0.05).

**Figure 4:**
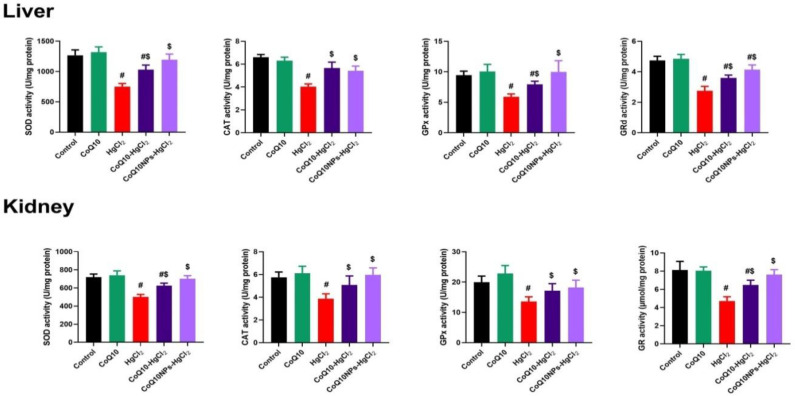
Palliative effects of coenzyme Q10-loaded albumin nanoparticles (CoQ10NPs, 10 mg/kg, orally, for 14 days) on hepatorenal antioxidant molecules (reduced glutathione (GSH), glutathione peroxidase (GPx), glutathione reductase (GR), and catalase (CAT)) upon exposure to mercuric chloride (HgCl_2_, 5 mg/kg, i.p., three times a week). Data are expressed as mean ± SD (n = 8/group). The symbol ^#^ means a significant difference from the control group, while ^$^ means a significant difference from the HgCl_2_ group at *p*-value < 0.05.

It is obvious from Figure 4 that HgCl_2_ toxicity suppressed antioxidant enzyme molecules (SOD, CAT, GR, and GPx) in both the liver and kidney compared to the control group (*p* < 0.05). However, the co-administration of CoQ10 and CoQ10NPs ameliorated the depleted antioxidant enzyme activities compared to those in HgCl_2_-subjected rats (*p* < 0.05). The co-treatment with CoQ10NPs restored hepatorenal SOD, CAT, GR, and GPx to their normal values, similar to those of the control rats (*p* < 0.05) (Figure 4).

### 3.4. Effect of CoQ10NPs on Hepatorenal Inflammatory Mediators upon HgCl_2_ Exposure

**Figure 5:**
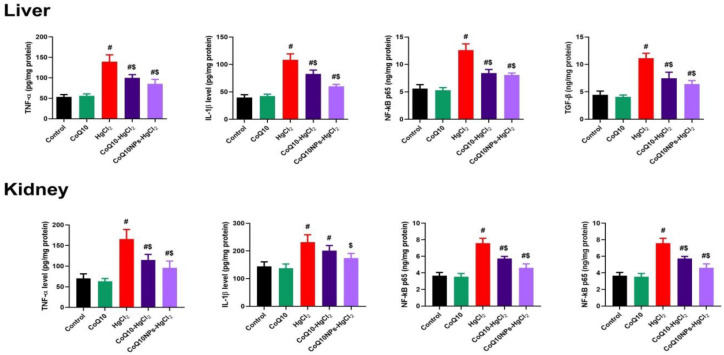
Palliative effects of coenzyme Q10-loaded albumin nanoparticles (CoQ10NPs, 10 mg/kg, orally, for 14 days) on hepatorenal inflammatory markers (tumor necrosis factor-α (TNF-α), interleukin 1 beta (IL-1β), nuclear factor Kappa (NF-ĸβ), and transforming growth factor β (TGF-β)) upon exposure to mercuric chloride (HgCl_2_, 5 mg/kg, i.p., three times a week). Data are expressed as mean ± SD (n = 8/group). The symbol ^#^ means a significant difference from the control group, while ^$^ means a significant difference from the HgCl_2_ group at *p*-value < 0.05.

Compared to the control group, HgCl_2_ exposure prompted inflammation in the liver and kidney, identified by a significant boost in the levels of inflammatory mediators (TNF-α, IL-1β, NF-κB, and TGF-β) (*p* < 0.05). Conversely, CoQ10 and CoQ10NP treatment diminished hepatorenal inflammation, as evidenced by a substantial drop in inflammatory markers following HgCl_2_ exposure, especially in the CoQ10NPs+HgCl_2_ group (*p* < 0.05) (Figure 5).

### 3.5. Effect of CoQ10NPs on Hepatic Alpha-Smooth Muscle Actin (α-SMA) Level upon HgCl_2_ Exposure

**Figure 6:**
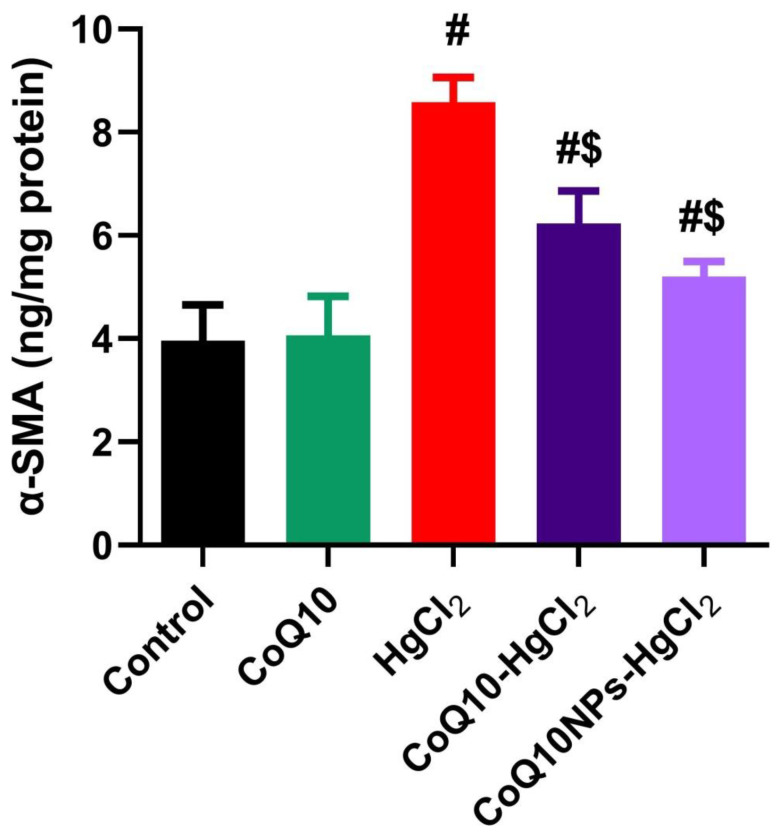
Palliative effects of coenzyme Q10-loaded albumin nanoparticles (CoQ10NPs, 10 mg/kg, orally, for 14 days) on the hepatic level of alpha-smooth muscle actin (α-SMA) upon exposure to mercuric chloride (HgCl_2_, 5 mg/kg, i.p., three times a week). Data are expressed as mean ± SD (n = 8/group). The symbol ^#^ means a significant difference from the control group, while ^$^ means a significant difference from the HgCl_2_ group at *p*-value < 0.05.

The α-SMA level was significantly raised in the livers of HgCl_2_-exposed rats compared to the control group. In comparison with the HgCl_2_ group, the hepatic α-SMA level was significantly ameliorated by CoQ10 and CoQ10NP treatments. The maximum effect was observed in the CoQ10NPs+HgCl_2_ group (*p* < 0.05) (Figure 6).

### 3.6. Effect of CoQ10NPs on Hepatorenal Apoptotic Markers upon HgCl_2_ Exposure

**Figure 7:**
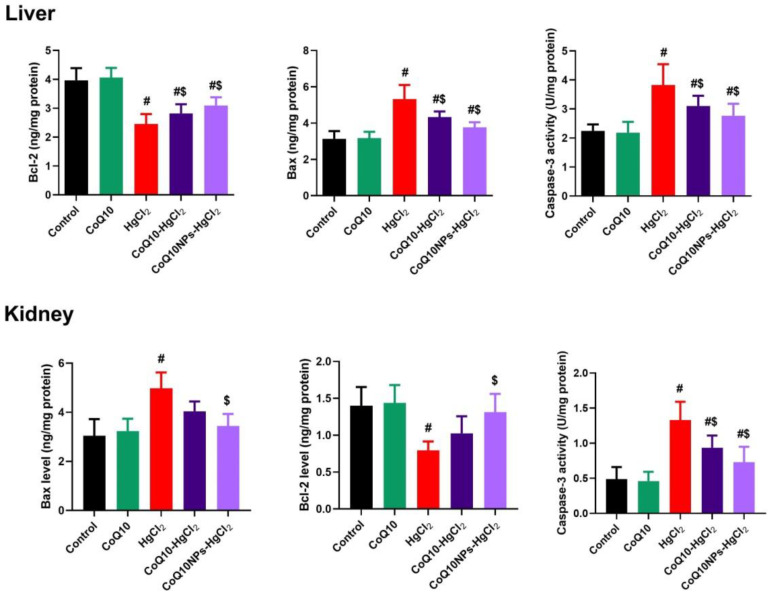
Palliative effects of coenzyme Q10-loaded albumin nanoparticles (CoQ10NPs, 10 mg/kg, orally, for 14 days) on hepatorenal apoptotic markers (Bcl-2-associated X protein (Bax), caspase-3, and B-cell lymphoma 2 (Bcl2)) upon exposure to mercuric chloride (HgCl_2_, 5 mg/kg, i.p., three times a week). Data are expressed as mean ± SD (n = 8/group). The symbol ^#^ means a significant difference from the control group, while ^$^ means a significant difference from the HgCl_2_ group at *p*-value < 0.05.

Pro-apoptotic (Bax and caspase-3) and anti-apoptotic (Bcl-2) proteins were measured to monitor the protective effects of CoQ10NPs against HgCl_2_-generated hepatorenal cell death, as illustrated in Figure 6. Compared to the control group, the HgCl_2_-intoxicated group showed significantly higher hepatic and renal levels of Bax and caspase-3 with lower Bcl-2 levels (*p* < 0.05). Interestingly, the simultaneous administration of CoQ10, either alone or conjugated with albumin nanoparticles, suppressed hepatic and renal tissue apoptosis through lowered Bax and caspase-3 levels, along with increased Bcl-2 levels, relative to the HgCl_2_-exposed group (*p* < 0.05) (Figure 7).

### 3.7. Effect of CoQ10NPs on Histological Alterations in Liver and Kidney upon HgCl_2_ Exposure

**Figure 8:**
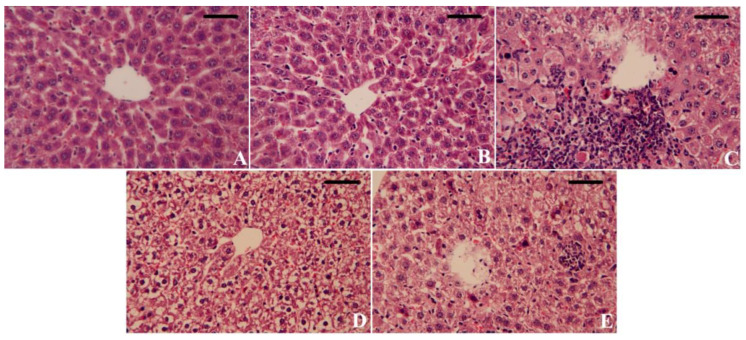
Palliative effects of coenzyme Q10-loaded albumin nanoparticles (CoQ10NPs, 10 mg/kg, orally, for 14 days) on histological changes in the liver upon exposure to mercuric chloride (HgCl_2_, 5 mg/kg, i.p., three times a week). Scale bar = 80 µm (400×). (**A**) Control group, (**B**) CoQ10 group, (**C**) HgCl_2_ group, (**D**) CoQ10-HgCl_2_ group, (**E**) CoQ10NPs-HgCl_2_ group.

The control and CoQ10 groups displayed the typical histological architectures of hepatocytes, central veins, and sinusoids (Figure 8A,B). The intraperitoneal injection of HgCl_2_ into rats caused congested blood vessels with pericellular leukocyte infiltration and focal inflammation in liver tissue. Many hepatocytes were suffering from pyknosis and apoptosis with an expanded area of necrosis (Figure 8C). CoQ10 administration was not able to protect the liver from HgCl_2_ toxicity according to histological results, where many hepatocytes displayed degeneration and apoptosis with activated Kuffer cells (Figure 8D). Interestingly, the administration of CoQ10NPs mitigated these effects. However, some focal inflammation and activated Kuffer cells were still seen (Figure 8E).

**Figure 9:**
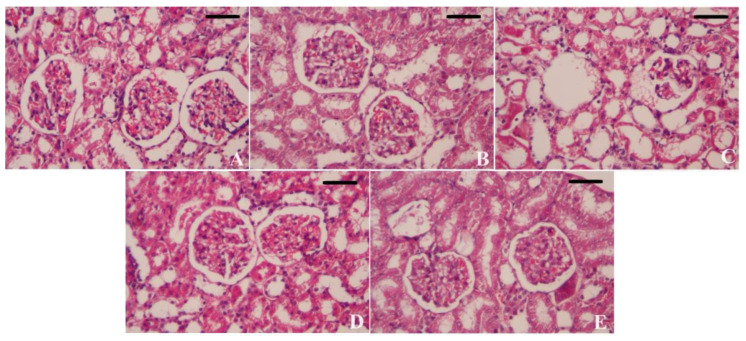
Palliative effects of coenzyme Q10-loaded albumin nanoparticles (CoQ10NPs, 10 mg/kg, orally, for 14 days) on histological changes in the kidney upon exposure to mercuric chloride (HgCl_2_, 5 mg/kg, i.p., three times a week). Scale bar = 80 µm (400×). (**A**) Control group, (**B**) CoQ10 group, (**C**) HgCl_2_ group, (**D**) CoQ10-HgCl_2_ group, (**E**) CoQ10NPs-HgCl_2_ group.

The kidneys of animals in the control and CoQ10-treated groups displayed typical histological images with a standard glomerular and tubular architecture (Figure 9A,B). Intoxication with HgCl_2_ induced glomerular degeneration and congestion and severe diffused tubular epithelial degeneration and vacuolation with tubular cast formation in the kidney and necrosis (Figure 9). In contrast, treating HgCl_2_-treated rats with CoQ10 or CoQ10NPs showed mild tubular degeneration and necrosis. The severity of congestion gradually decreased and remained normal (Figure 9D,E). However, CoQ10NPs were revealed to have superior protection to CoQ10 per se.

## 4. Discussion

For many years, mercury has been known to be a very hazardous metal for humans [4]. Mercury exposure is frequently brought on by the environment and common occupational or dental amalgams [2]. It is widely known that inorganic mercury has an affinity for endogenous biomolecules’ SH groups. As a result, it can be connected to proteins that contain thiols and low-molecular-weight thiols (cysteine and glutathione) [42]. Additionally, mercury can generate free radicals that oxidize lipids, proteins, and DNA [43]. HgCl_2_ generates hepatorenal toxicity via different pathways, including inflammation, oxidative stress, and apoptosis [14,44].

According to our results, following HgCl_2_ injection, the liver and renal functions were adversely affected, leading to hepatorenal dysfunction, which was demonstrated by significant increases in AST, ALT, and ALP enzyme activities, as well as urea, creatinine, and Kim-1 levels. Similar results were previously reported by Joshi et al. [45], Elblehi et al. [46], and Nabil et al. [14]. According to our histopathological investigations (Figure 8 and Figure 9), these increases in liver enzyme activities are usually related to HgCl_2_’s harmful actions on the cell membranes of hepatocytes, which may cause necrotic and hepatic lesions [47,48]. The elevated levels of urea, creatinine, and Kim-1 in the serum of rats treated with HgCl_2_ reflect cumulative renal insufficiency [49].

CoQ10, a strong antioxidant, dramatically lowers liver enzyme activity following hepatic inflammation and degeneration caused by CCl_4_ intoxication [50,51]. Based on our findings, the co-administration of CoQ10, either alone or loaded with albumin nanoparticles, abrogated HgCl_2_-induced hepatorenal damage, as demonstrated by the reduced AST, ALT, creatinine, urea, and Kim-1 levels. The histological findings are consistent with the biochemical data, indicating that CoQ10 and CoQ10NP supplementation had a significant protective effect against HgCl_2_-induced hepatorenal damage. Additionally, following lead exposure, CoQ10 reversed the raised levels of serum urea and creatinine, demonstrating that it has a renal protective effect by preserving membrane integrity and preventing the leaking of these nitrogenous indicators into the bloodstream [52,53]. In other experimental models, CoQ10 decreased the increased levels of ALT, AST, ALP, creatinine, and urea mediated by piroxicam [54] and arsenic toxicity [55].

Lipid, protein, and DNA damage occurs through oxidative damage, which predominantly results from the generation of reactive oxygen species, such as the superoxide anion and peroxides [42]. It can result in the loss of enzymatic activity and structural integrity and trigger inflammatory processes [56]. In the current study, antioxidant molecule activities (SOD, GPx, GR, and CAT) as well as GSH levels were all significantly decreased in both the liver and kidneys of rats exposed to HgCl_2_. Also, oxidative stress indices (MDA and NO) were markedly elevated. Numerous earlier studies support the idea that HgCl_2_ may harm hepatic and renal cells through oxidative stress [9,57,58,59]. The oxidative stress caused by mercury in the liver and kidney tissues is attributed to alterations in the structural integrity of the mitochondrial inner membrane, the depletion of the levels of mitochondrial glutathione, and an increase in the production of hydrogen peroxide by the mitochondrial electron transport chain [46,60,61]. The increased production of free radicals due to HgCl_2_ toxicity may result in raised levels of MDA and NO [43,57]. Furthermore, the observed reductions in SOD, CAT, GPx, and GR enzymes may be caused by the inhibition of these enzymes by high levels of free radicals such as H_2_O_2_ [42,58]. In addition, mercury blocks the function of GSH as a result of its affinity for the thiol groups present in the structure of GSH [59].

CoQ10 is regarded as a special mitochondrial antioxidant, as it can reduce the production of excess NO, protect against LPO, protein oxidation, and DNA damage, and decrease the expression of NADPH oxidase, which is a significant generator of superoxide anions [54]. It has been reported that CoQ10 supplementation balanced the redox homeostasis in the renal tissue and activated Nrf2/HO-1 signaling in response to renal injury mediated by lead acetate in rats [17]. In addition, CoQ10 administration diminished MDA and NO production while increasing GSH content and the activity of GPx1, SOD, and CAT following doxorubicin-induced oxidative damage in rats [60]. Our findings also showed that rats pretreated with CoQ10 or CoQ10NP before receiving the HgCl_2_ injection showed a statistically significant improvement in the oxidant/antioxidant balance, demonstrated by an increase in the antioxidant enzymes with a decrease in oxidative stress indicators (LPO and NO). Relative to Q10 alone, CoQ10NPs produced substantially lower levels of oxidative stress markers and higher quantities of antioxidant molecules.

It is believed that inflammation plays a key role in the hepatotoxicity caused by HgCl_2_ and is frequently associated with increased ROS production and oxidative stress [12]. The inflammatory pathway activated by NF-κB transcription is a crucial process that has been implicated in the HgCl_2_-induced inflammatory response through enhanced interleukin production, which eventually results in hepatic and renal cell death [61]. Consequently, the current study demonstrated that rats exposed to HgCl_2_ had significantly higher levels of TNF-α, IL-1β, NF-κB, and TGF-β gene expression than control rats, indicating hepatorenal inflammation and fibrosis. Prior studies have shown that HgCl_2_ exposure increased the generation of reactive free radicals, which, in turn, increased the release of inflammatory cytokines (TNF-α, IL-1β, IL-6) and NF-κB [62,63]. Transforming growth factor (TGF-β) was thought to be a crucial component of hepatic and renal fibrogenesis [64,65]. Moreover, the induction of T-cell autoimmune syndrome by mercury resulted in a significant increase in TGF-β production and various cytokines, consequently causing collagen deposition [66]. According to earlier research, rats treated with HgCl_2_ had significantly higher levels of renal and hepatic TGF-β compared to control rats [14,67,68]. Our study also showed that HgCl_2_ toxicity statistically increases hepatic α-SMA compared with the control group. This is considered evidence of tissue fibrosis, which is confirmed by the histological changes in the liver that were recorded in our results [69]. Similar results previously revealed that HgCl_2_ significantly increases renal fibrosis and α-SMA-positive cells [70,71].

In this context, AL-Megrin et al. [17] found that CoQ10 reduced NF-κB transactivation, which subsequently decreased the expression of pro-inflammatory cytokines. Furthermore, CoQ10 inhibited the migration of leukocytes, including monocytes and macrophages, which have a vital role in the production of pro-inflammatory cytokines [70]. Our data also indicated that CoQ10 and CoQ10NPs inhibited the robust elevation of pro-inflammatory cytokines and fibrotic markers in the hepatic and renal tissues of HgCl_2_ acetate-intoxicated rats. Similarly, it has been reported that coenzyme Q10 could reduce inflammation and fibrosis linked to radiation enteropathy by inhibiting the NF-kB/TGF-β/MMP-9 pathway [71].

The relationship between the pro-apoptotic protein Bax and the anti-apoptotic protein Bcl-2 can regulate the cell apoptotic pathway [72]. Additionally, the mitochondrial (intrinsic) pathway is also one of the crucial apoptotic mechanisms, which is characterized by elevated amounts of cytochrome C in the cytoplasm as a result of the disturbed Bax/Bcl-2 balance [73]. Caspase-3 is activated when cytochrome c levels are elevated, which encourages apoptosis. Through an increase in intrinsic pathways, caspase-3 becomes more active and triggers apoptosis [74]. Recent reports confirmed that HgCl_2_ had an apoptotic impact on both the liver and kidney by increasing Bax and caspase-3, along with decreasing Bcl-2 levels [61,63]. This was corroborated by our findings, which showed that exposure to HgCl_2_ caused liver and kidney cell death due to the overactivation of Bax and caspase-3 while suppressing the Bcl-2 protein.

The potential anti-apoptotic properties of CoQ10 are realized through increased levels of the anti-apoptotic protein (Bcl-2), reduced oxidative stress, and consequently, the prevention of mitochondria from releasing cytochrome c [75]. Our findings revealed that treatment with CoQ10 and CoQ10NPs significantly restored HgCl_2_-mediated changes in Bax, caspase 3, and Bcl-2 levels, demonstrating their anti-apoptotic activity. These data are consistent with the previous study by Abdeen et al. [54], who confirmed the anti-apoptotic effect of CoQ10 against piroxicam-induced hepatorenal toxicity. Moreover, the co-administration of CoQ10 suppressed the elevation of Bax and caspase-3, as well as the reduction in Bcl-2 levels in the renal tissue [17].

Interestingly, the utilization of antioxidant medication showed greater hepato-protectivity when it was encapsulated in albumin nanoparticles. This could be explained by the effective localization, ingestion, and endocytic absorption of albumin nanoparticles via several mechanisms [76]. The presence of albumin may potentially have a synergistic antioxidant impact. Both the direct and indirect antioxidant effects of serum albumin have been demonstrated. It works directly as a free radical scavenger because the free thiol groups in the Cys-34 residue are frequently able to trap various types of ROS and the presence of six easily oxidized methionine groups [77]. Serum albumin can also have an indirect effect by binding to free metals like iron and copper that promote the generation of aggressive ROS and bilirubin, which inhibits lipid peroxidation [78]. Previous reports demonstrated that the conjugation of bovine serum albumin with carvacrol reduced the levels of NO and an inflammation-promoting cytokine (IL-17) in arthritic rats [79]. Similarly, our findings emphasized that CoQ10 is more effective when it is enclosed in albumin nanocapsules. Compared to HgCl_2_-intoxicated rats treated with CoQ10 alone, those treated with CoQ10-loaded albumin showed greater mitigation of the intensity of hepatotoxicity, oxidative stress, inflammation, and apoptosis. Therefore, CoQ10 encapsulated with albumin nanoparticles (CoQ10NPs) is suggested to be a valuable approach for improving the therapeutic applications of CoQ10.

## 5. Conclusions

Collectively, this study suggests that the encapsulation of CoQ10 into albumin nanoparticles (CoQ10NPs) led to the significantly enhanced bioavailability of CoQ10 and hepatorenal protection in HgCl_2_-intoxicated rats. CoQ10NPs alleviated oxidative stress, inflammation, and apoptosis, which are implicated in the pathophysiology of HgCl_2_ poisoning. Pretreatment with CoQ10 or CoQ10NPs stimulated the activity of antioxidant enzymes and decreased inflammatory cytokines in the HgCl_2_-exposed rats. A notable hepatorenal protective effect against apoptosis caused by HgCl_2_ was also imparted by CoQ10NPs. The protective potential of CoQ10 and CoQ10NPs was impacted by the blockage of NF-kB/TGF-β signaling. The present study is also of great clinical importance, as free CoQ10 had a weaker ameliorating effect due to its poor water solubility. The improved bioavailability of CoQ10NPs could be explained by the increased solubilization of CoQ10 when encapsulated into albumin nanoparticles. Our study reveals that the utilization of albumin nanoparticles to encapsulate CoQ10 is an effective strategy for delivering CoQ10 to tissues. However, further in vitro and in vivo studies will be performed to investigate the digestion of the nanoparticles and the content of CoQ10 to ensure the structural integrity and maintenance of CoQ10NPs under gastric digestion conditions to deliver them to the intestine for absorption. Also, the bioaccessibility of CoQ10NPs will be studied to ensure the improved effect of CoQ10NPs compared to the CoQ10 free form with gastrointestinal tract digestion.

## Data Availability

All data generated or analyzed during this study are included in this published article.

## Supplementary Materials

The following supporting information can be downloaded at https://www.mdpi.com/article/10.3390/biomedicines11113054/s1: Table S1: FT-IR peak table results of CoQ10 and CoQ10NPs.

## References

[1] Al Olayan E.M., Aloufi A.S., AlAmri O.D., El-Habit O.H., Abdel Moneim A.E. (2020). Protocatechuic acid mitigates cadmium-induced neurotoxicity in rats: Role of oxidative stress, inflammation and apoptosis. Sci. Total Environ..

[2] Othman M.S., Safwat G., Aboulkhair M., Abdel Moneim A.E. (2014). The potential effect of berberine in mercury-induced hepatorenal toxicity in albino rats. Food Chem. Toxicol..

[3] Goudarzi M., Kalantar M., Kalantar H. (2017). The hepatoprotective effect of gallic acid on mercuric chloride-induced liver damage in rats. Jundishapur J. Nat. Pharm. Prod..

[4] Almeer R.S., Albasher G., Kassab R.B., Ibrahim S.R., Alotibi F., Alarifi S., Ali D., Alkahtani S., Abdel Moneim A.E. (2020). Ziziphus spina-christi leaf extract attenuates mercury chloride-induced testicular dysfunction in rats. Environ. Sci. Pollut. Res. Int..

[5] Boujbiha M.A., Hamden K., Guermazi F., Bouslama A., Omezzine A., Kammoun A., El Feki A. (2009). Testicular toxicity in mercuric chloride treated rats: Association with oxidative stress. Reprod. Toxicol..

[6] Moneim A.E.A. (2015). The neuroprotective effect of berberine in mercury-induced neurotoxicity in rats. Metab. Brain Dis..

[7] Rozgaj R., Kašuba V., Blanuša M. (2005). Mercury chloride genotoxicity in rats following oral exposure, evaluated by comet assay and micronucleus test. Arh. Za Hig. Rada I Toksikol..

[8] Sharma M.K., Sharma A., Kumar A., Kumar M. (2007). Evaluation of protective efficacy of Spirulina fusiformis against mercury induced nephrotoxicity in Swiss albino mice. Food Chem. Toxicol..

[9] Durak D., Kalender S., Uzun F.G., Kalender Y. (2010). Mercury chloride-induced oxidative stress in human erythrocytes and the effect of vitamins C and E in vitro. Afr. J. Biotechnol..

[10] Boujbiha M.A.M., Hamden K., Guermazi F., Bouslama A., Omezzine A., El Feki A. (2011). Impairment of spermatogenesis in rats by mercuric chloride: Involvement of low 17β-estradiol level in induction of acute oxidative stress. Biol. Trace Elem. Res..

[11] Vijayaprakash S., Langeswaran K., Kumar S.G., Revathy R., Balasubramanian M.P. (2013). Nephro-protective significance of kaempferol on mercuric chloride induced toxicity in Wistar albino rats. Biomed. Aging Pathol..

[12] Zhang H., Tan X., Yang D., Lu J., Liu B., Baiyun R., Zhang Z. (2017). Dietary luteolin attenuates chronic liver injury induced by mercuric chloride via the Nrf2/NF-κB/P53 signaling pathway in rats. Oncotarget.

[13] Ansar S., Iqbal M. (2016). Protective effect of diallylsulphide against mercuric chloride-induced hepatic injury in rats. Hum. Exp. Toxicol..

[14] Nabil A., Elshemy M.M., Asem M., Gomaa H.F. (2020). Protective effect of DPPD on mercury chloride-induced Hepatorenal toxicity in rats. J. Toxicol..

[15] Suárez-Rivero J.M., Pastor-Maldonado C.J., Povea-Cabello S., Álvarez-Córdoba M., Villalón-García I., Munuera-Cabeza M., Suárez-Carrillo A., Talaverón-Rey M., Sánchez-Alcázar J.A. (2021). Coenzyme Q10 analogues: Benefits and challenges for therapeutics. Antioxidants.

[16] Parmar S.S., Jaiwal A., Dhankher O.P., Jaiwal P.K. (2015). Coenzyme Q10 production in plants: Current status and future prospects. Crit. Rev. Biotechnol..

[17] Al-Megrin W.A., Soliman D., Kassab R.B., Metwally D.M., Moneim A.E.A., El-Khadragy M.F. (2020). Coenzyme Q10 activates the antioxidant machinery and inhibits the inflammatory and apoptotic cascades against lead acetate-induced renal injury in rats. Front. Physiol..

[18] Yousef S., Omar A., Fahad A.A., Abdel Moneim A.E., Metwally D.M., El-khadragy M.F., Kassab R.B. (2019). The neuroprotective role of coenzyme Q10 against lead acetate-induced neurotoxicity is mediated by antioxidant, anti-inflammatory and anti-apoptotic activities. Int. J. Environ. Res. Public Health.

[19] Sohet F.M., Delzenne N.M. (2012). Is there a place for coenzyme Q in the management of metabolic disorders associated with obesity?. Nutr. Rev..

[20] Noh Y., Kim K., Shim M., Choi S., Choi S., Ellisman M., Weinreb R., Perkins G., Ju W. (2013). Inhibition of oxidative stress by coenzyme Q10 increases mitochondrial mass and improves bioenergetic function in optic nerve head astrocytes. Cell Death Dis..

[21] Blatt T., Littarru G.P. (2011). Biochemical rationale and experimental data on the antiaging properties of CoQ10 at skin level. Biofactors.

[22] Patil T.S., Deshpande A.S. (2018). Nanostructured lipid carriers-based drug delivery for treating various lung diseases: A state-of-the-art review. Int. J. Pharm..

[23] Zhang Y., Sun T., Jiang C. (2018). Biomacromolecules as carriers in drug delivery and tissue engineering. Acta Pharm. Sin. B.

[24] Kratz F., Fichtner I., Beyer U., Schumacher P., Roth T., Fiebig H., Unger C. (1997). Antitumour activity of acid labile transferrin and albumin doxorubicin conjugates in in vitro and in vivo human tumour xenograft models. Eur. J. Cancer.

[25] Mariam J., Sivakami S., Dongre P.M. (2016). Albumin corona on nanoparticles—A strategic approach in drug delivery. Drug Deliv..

[26] Kunde S.S., Wairkar S. (2022). Targeted delivery of albumin nanoparticles for breast cancer: A review. Colloids Surf. B Biointerfaces.

[27] Pinto S., Gaspar M.M., Ascensão L., Faísca P., Reis C.P., Pacheco R. (2022). Nanoformulation of Seaweed Eisenia bicyclis in Albumin Nanoparticles Targeting Cardiovascular Diseases: In Vitro and In Vivo Evaluation. Mar. Drugs.

[28] Kianfar E. (2021). Protein nanoparticles in drug delivery: Animal protein, plant proteins and protein cages, albumin nanoparticles. J. Nanobiotechnol..

[29] Elzoghby A.O., Samy W.M., Elgindy N.A. (2012). Protein-based nanocarriers as promising drug and gene delivery systems. J. Control. Release.

[30] Fatima S., Suhail N., Alrashed M., Wasi S., Aljaser F.S., AlSubki R.A., Alsharidah A.S., Banu N. (2021). Epigallocatechin gallate and coenzyme Q10 attenuate cisplatin-induced hepatotoxicity in rats via targeting mitochondrial stress and apoptosis. J. Biochem. Mol. Toxicol..

[31] El-Desoky G.E., Bashandy S.A., Alhazza I.M., Al-Othman Z.A., Aboul-Soud M.A., Yusuf K. (2013). Improvement of mercuric chloride-induced testis injuries and sperm quality deteriorations by Spirulina platensis in rats. PLoS ONE.

[32] El-Sheikh A.A., Morsy M.A., Mahmoud M.M., Rifaai R.A., Abdelrahman A.M. (2012). Effect of coenzyme-Q10 on doxorubicin-induced nephrotoxicity in rats. Adv. Pharmacol. Pharm. Sci..

[33] Jithan A., Madhavi K., Madhavi M., Prabhakar K. (2011). Preparation and characterization of albumin nanoparticles encapsulating curcumin intended for the treatment of breast cancer. Int. J. Pharm. Investig..

[34] Ohkawa H., Ohishi N., Yagi K. (1979). Assay for lipid peroxides in animal tissues by thiobarbituric acid reaction. Anal. Biochem..

[35] Green L.C., Wagner D.A., Glogowski J., Skipper P.L., Wishnok J.S., Tannenbaum S.R. (1982). Analysis of nitrate, nitrite, and [15N]nitrate in biological fluids. Anal. Biochem..

[36] Nishikimi M., Appaji N., Yagi K. (1972). The occurrence of superoxide anion in the reaction of reduced phenazine methosulfate and molecular oxygen. Biochem. Biophys. Res. Commun..

[37] Ellman G.L. (1959). Tissue sulfhydryl groups. Arch. Biochem. Biophys..

[38] Paglia D.E., Valentine W.N. (1967). Studies on the quantitative and qualitative characterization of erythrocyte glutathione peroxidase. J. Lab. Clin. Med..

[39] Smith I.K., Vierheller T.L., Thorne C.A. (1988). Assay of glutathione reductase in crude tissue homogenates using 5,5′-dithiobis (2-nitrobenzoic acid). Anal. Biochem..

[40] Aebi H. (1984). Catalase in vitro. Methods Enzym..

[41] Bradford M.M. (1976). A rapid and sensitive method for the quantitation of microgram quantities of protein utilizing the principle of protein-dye binding. Anal. Biochem..

[42] Kalender S., Uzun F.G., Demir F., Uzunhisarcıklı M., Aslanturk A. (2013). Mercuric chloride-induced testicular toxicity in rats and the protective role of sodium selenite and vitamin E. Food Chem. Toxicol..

[43] Su L., Wang M., Yin S.-T., Wang H.-L., Chen L., Sun L.-G., Ruan D.-Y. (2008). The interaction of selenium and mercury in the accumulations and oxidative stress of rat tissues. Ecotoxicol. Environ. Saf..

[44] Yadav H.N., Sharma U.S., Singh S., Gupta Y.K. (2019). Effect of Tribulus terrestris in mercuric chloride-induced renal accumulation of mercury and nephrotoxicity in rat. J. Adv. Pharm. Technol. Res..

[45] Joshi D., Mittal D.K., Shukla S., Srivastav S.K., Dixit V.A. (2017). Curcuma longa Linn. extract and curcumin protect CYP 2E1 enzymatic activity against mercuric chloride-induced hepatotoxicity and oxidative stress: A protective approach. Exp. Toxicol. Pathol..

[46] Elblehi S.S., Hafez M.H., El-Sayed Y.S. (2019). L-α-Phosphatidylcholine attenuates mercury-induced hepato-renal damage through suppressing oxidative stress and inflammation. Environ. Sci. Pollut. Res..

[47] Uzunhisarcikli M., Aslanturk A., Kalender S., Apaydin F.G., Bas H. (2016). Mercuric chloride induced hepatotoxic and hematologic changes in rats: The protective effects of sodium selenite and vitamin E. Toxicol. Ind. Health.

[48] Salman M.M., Kotb A.M., Haridy M.A., Hammad S. (2016). Hepato-and nephroprotective effects of bradykinin potentiating factor from scorpion (Buthus occitanus) venom on mercuric chloride-treated rats. EXCLI J..

[49] Oriquat G.A., Saleem T.H., Naik R.R., Moussa S.Z., Al-Gindy R.M. (2012). A Sub-Chronic Toxicity Study of Mercuric Chloride in the Rat. Jordan J. Biol. Sci..

[50] Al-Rekabi B.K.K., Al-Diwan M.A., Sawad A.A. (2019). The protective role of CoQ10 and DHEA and their combination on CCl4 induced liver injury in adult male rats (*Rattus norvegicus*). J. Biosci. Appl. Res..

[51] Ali S.A., Faddah L., Abdel-Baky A., Bayoumi A. (2010). Protective effect of L-carnitine and coenzyme Q10 on CCl4-induced liver injury in rats. Sci. Pharm..

[52] Ustuner M.A., Kaman D., Colakoglu N. (2017). Effects of benfotiamine and coenzyme Q10 on kidney damage induced gentamicin. Tissue Cell.

[53] Ki Y., Kim W., Kim Y.H., Kim D., Bae J.S., Park D., Jeon H., Lee J.H., Lee J., Nam J. (2017). Effect of coenzyme Q10 on radiation nephropathy in rats. J. Korean Med. Sci..

[54] Abdeen A., Abdelkader A., Elgazzar D., Aboubakr M., Abdulah O.A., Shoghy K., Abdel-Daim M., El-Serehy H.A., Najda A., El-Mleeh A. (2020). Coenzyme Q10 supplementation mitigates piroxicam-induced oxidative injury and apoptotic pathways in the stomach, liver, and kidney. Biomed. Pharmacother..

[55] Mwaeni V.K., Nyariki J.N., Jillani N., Omwenga G., Ngugi M., Isaac A.O. (2021). Coenzyme Q10 protected against arsenite and enhanced the capacity of 2,3-dimercaptosuccinic acid to ameliorate arsenite-induced toxicity in mice. BMC Pharmacol. Toxicol..

[56] Özyurt H., Söğüt S., Yıldırım Z., Kart L., Iraz M., Armutçu F., Temel İ., Özen S., Uzun A., Akyol Ö. (2004). Inhibitory effect of caffeic acid phenethyl ester on bleomycine-induced lung fibrosis in rats. Clin. Chim. Acta.

[57] Aslanturk A., Uzunhisarcikli M., Kalender S., Demir F. (2014). Sodium selenite and vitamin E in preventing mercuric chloride induced renal toxicity in rats. Food Chem. Toxicol..

[58] Rao M.V., Chhunchha B. (2010). Protective role of melatonin against the mercury induced oxidative stress in the rat thyroid. Food Chem. Toxicol..

[59] Hosseini A., Rajabian A., Fanoudi S., Farzadnia M., Boroushaki M.T. (2018). Protective effect of Rheum turkestanicum root against mercuric chloride-induced hepatorenal toxicity in rats. Avicenna J. Phytomed..

[60] Mustafa H.N., Hegazy G.A., El Awdan S.A., AbdelBaset M. (2017). Protective role of CoQ10 or L-carnitine on the integrity of the myocardium in doxorubicin induced toxicity. Tissue Cell.

[61] Caglayan C., Kandemir F.M., Darendelioğlu E., Yıldırım S., Kucukler S., Dortbudak M.B. (2019). Rutin ameliorates mercuric chloride-induced hepatotoxicity in rats via interfering with oxidative stress, inflammation and apoptosis. J. Trace Elem. Med. Biol..

[62] Li S., Wang X., Xiao Y., Wang Y., Wan Y., Li X., Li Q., Tang X., Cai D., Ran B. (2021). Curcumin ameliorates mercuric chloride-induced liver injury via modulating cytochrome P450 signaling and Nrf2/HO-1 pathway. Ecotoxicol. Environ. Saf..

[63] Yang D., Tan X., Lv Z., Liu B., Baiyun R., Lu J., Zhang Z. (2016). Regulation of Sirt1/Nrf2/TNF-α signaling pathway by luteolin is critical to attenuate acute mercuric chloride exposure induced hepatotoxicity. Sci. Rep..

[64] Kao Y.-H., Chen C.-L., Jawan B., Chung Y.-H., Sun C.-K., Kuo S.-M., Hu T.-H., Lin Y.-C., Chan H.-H., Cheng K.-H. (2010). Upregulation of hepatoma-derived growth factor is involved in murine hepatic fibrogenesis. J. Hepatol..

[65] Suzuki K., Wang R., Kubota H., Shibuya H., Saegusa J., Sato T. (2005). Kinetics of biglycan, decorin and thrombospondin-1 in mercuric chloride-induced renal tubulointerstitial fibrosis. Exp. Mol. Pathol..

[66] Hemdan N., Lehmann I., Wichmann G., Lehmann J., Emmrich F., Sack U. (2007). Immunomodulation by mercuric chloride in vitro: Application of different cell activation pathways. Clin. Exp. Immunol..

[67] Karabulut D., Akin A.T., Unsal M., Lekesizcan A., Ozyazgan T.M., Keti D.B., Yakan B., Ekebas G. (2021). L-Carnitine ameliorates the liver by regulating alpha-SMA, iNOS, HSP90, HIF-1alpha, and RIP1 expressions of CCL4-toxic rats. Iran. J. Basic Med. Sci..

[68] Elshemy M.M., AbdEl-Mejied A.E., Zahran F., Omran M.M., Nabil A. (2018). DPPD ameliorates renal fibrosis induced by HgCl2 in rats. Biosci. Res..

[69] Tao Y., Wang Q., Yuan J., Shen L., Liu C. (2011). Effects of vitamin E on mercuric chloride-induced renal interstitial fibrosis in rats and the antioxidative mechanism. J. Chin. Integr. Med..

[70] Lavinya B.U., Bardhan I., Prince S.E. (2016). Efficacy of CoenzymeQ10 in inhibiting monosodium urate crystal-induced inflammation in rats. Eur. J. Pharmacol..

[71] Mohamed H.A., Said R.S. (2021). Coenzyme Q10 attenuates inflammation and fibrosis implicated in radiation enteropathy through suppression of NF-kB/TGF-β/MMP-9 pathways. Int. Immunopharmacol..

[72] Abdel Moneim A.E. (2016). Indigofera oblongifolia prevents lead acetate-induced hepatotoxicity, oxidative stress, fibrosis and apoptosis in rats. PLoS ONE.

[73] Izuta H., Shimazawa M., Tazawa S., Araki Y., Mishima S., Hara H. (2008). Protective effects of Chinese propolis and its component, chrysin, against neuronal cell death via inhibition of mitochondrial apoptosis pathway in SH-SY5Y cells. J. Agric. Food Chem..

[74] Darendelioglu E., Aykutoglu G., Tartik M., Baydas G. (2016). Turkish propolis protects human endothelial cells in vitro from homocysteine-induced apoptosis. Acta Histochem..

[75] Sumi K., Okura T., Fujioka Y., Kato M., Imamura T., Taniguchi S.-i., Yamamoto K. (2018). Coenzyme Q10 suppresses apoptosis of mouse pancreatic β-cell line MIN6. Diabetol. Metab. Syndr..

[76] Mo Y., Barnett M.E., Takemoto D., Davidson H., Kompella U.B. (2007). Human serum albumin nanoparticles for efficient delivery of Cu, Zn superoxide dismutase gene. Mol. Vis..

[77] Zaher S., Soliman M.E., Elsabahy M., Hathout R.M. (2022). Sesamol loaded albumin nanoparticles: A boosted protective property in animal models of oxidative stress. Pharmaceuticals.

[78] Arroyo V., García-Martinez R., Salvatella X. (2014). Human serum albumin, systemic inflammation, and cirrhosis. J. Hepatol..

[79] Gholijani N., Abolmaali S.-S., Kalantar K., Ravanrooy M.-H. (2020). Therapeutic effect of carvacrol-loaded albumin nanoparticles on arthritic rats. Iran. J. Pharm. Res. IJPR.

