# Supplementary material for: Coenzyme Q10-Loaded Albumin Nanoparticles Protect against Redox Imbalance and Inflammatory, Apoptotic, and Histopathological Alterations in Mercuric Chloride-Induced Hepatorenal Toxicity in Rats
Source: Biomedicines. 2023 Nov 14;11(11):3054. doi: 10.3390/biomedicines11113054 (PMC10669886; doi:10.3390/biomedicines11113054)
Supplement: Supplementary file 1 [file biomedicines-11-03054-s001.zip › biomedicines-2673402-supplementary.pdf]

Supplementary data; Table S1: FT-IR peak table results of CoQ10 and CoQ10NPs.

CoQ10			CoQ10NPs		
**Peak Number**	**X (cm^{-1})**	**Y (%T)**	**Peak Number**	**X (cm^{-1})**	**Y (%T)**
1	3324.27	56.60	1	3324.33	57.94
2	2118.14	96.24	2	2117.33	96.26
3	1634.72	72.48	3	1634.00	74.06
4	1334.35	89.30	4	1334.29	89.86
			**5***	**1235.03**	**90.11**
5	1151.74	87.33	6	1151.94	88.35
6	1077.18	85.24	7	1077.14	86.69
7	1014.31	76.58	8	1016.20	78.57
8	686.26	47.73	9	686.50	48.59
9	643.86	43.30	10	643.84	44.31
10	625.91	42.10	11	626.27	43.17
11	566.97	36.63	12	567.49	37.75
12	514.48	34.34	13	515.50	35.49
13	498.88	33.85	14	499.53	34.95
14	463.11	33.69	15	463.07	34.94

*Red color indicates the new formed bond between CoQ10 and albumin.
